# LanCL1 protects prostate cancer cells from oxidative stress via suppression of JNK pathway

**DOI:** 10.1038/s41419-017-0207-0

**Published:** 2018-02-07

**Authors:** Jianqing Wang, Qianyi Xiao, Xu Chen, Shijun Tong, Jianliang Sun, Ruitu Lv, Siqing Wang, Yuancheng Gou, Li Tan, Jianfeng Xu, Caibin Fan, Guanxiong Ding

**Affiliations:** 1 Department of Urology, The Affiliated Suzhou Hospital of Nanjing Medical University, Suzhou Municipal Hospital, 26 Daoqian Rd, Suzhou, 215000 China; 2 Key Laboratory of Public Health Safety, Ministry of Education, School of Public Health, Fudan University, Shanghai, China; 3 Institutes of Biomedical Sciences, Fudan University, Shanghai, China; 4 Department of Urology, Huashan Hospital, Fudan University, Shanghai, China

## Abstract

Prostate cancer (PCa) is the most commonly diagnosed malignancy in male. Numerous studies have focused on the molecular mechanisms of carcinogenesis and progression, aiming at developing new therapeutic strategies. Here we describe Lanthionine synthase C-like protein 1 (LanCL1), a member of the LanCL family, is a potential prostate cancer susceptibility gene. LanCL1 promotes prostate cancer cell proliferation and helps protect cells from damage caused by oxidative stress. Suppression of LanCL1 by siRNA results in increased cancer cell apoptosis. Clinical data also indicate that LanCL1 upregulation in human prostate cancers correlates with tumor progression. Finally, we demonstrate that LanCL1 plays such important role through inhibiting JNK pathway. Altogether, our results suggest that LanCL1 protects cells from oxidative stress, and promotes cell proliferation. LanCL1 reduces cell death via suppression of JNK signaling pathway.

## Introduction

Prostate cancer is the most commonly diagnosed non-cutaneous malignancy and the second leading cause of cancer-related death among men in the developed world^1,2^. Up to now, we still know very little about the molecular mechanisms of prostate cancer development and progression. Therefore, further understanding of the precise molecular mechanisms of the disease is necessary to develop some new effective strategies for treatment^3^.

Lanthionine synthetase C-like protein 1 (LanCL1, also known as P40 or GRP69A)^4^ is a mammalian member of the LanC-like protein superfamily encompassing a highly divergent group of peptide-modifying enzymes present in plants and bacteria (LanCs). Previous studies have shown that human LanCL1 protein binds zinc ion and GSH, and is essential for mitigating neuronal oxidative stress during normal postnatal development. In addition, LanCL1 catalyzes the formation of thioether products, and protects neurons from oxidative stress^5–7^. There have been reports that verified the relationship between LanCL1 and cancer. LanCL1 can serve as a potential marker of senescence, and the expression of LanCL1 correlates with increased survival in breast cancer^8^. By deeply querying online data sets, we found that LanCL1 expresses higher in tumor tissues, but found no reports that explain the role of LanCL1 in the initiation and progression of prostate cancer.

Prostate cancer development is a complex process involving uncontrolled proliferation, migration, and survival at the secondary site. Moreover, cancer cells still have the ability to protect themselves from apoptosis caused by extracellular environment, including oxidative stress and other damage^9,10^. The role of ROS and oxidative stress in prostate cancer initiation, progression is important and complicated. ROS contributes to cancerogenesis, progression and even the resistance to chemotherapeutic drugs, while high level of ROS induces cell death. Previous studies have shown us that LanCL1 involves in cellular process related to ROS and oxidative stress, thus making us interest in its role in prostate cancer. In this study, we demonstrated that LanCL1 highly expresses in prostate cancer tissues, TRAMP prostate cancer tissue, and especially in high-grade tumor tissues and metastatic prostate cancer cell lines. We found that LanCL1 promotes prostate cancer cell proliferation and protects cells from oxidative damage. LanCL1 does not mitigate oxidative level in cancer cells, but inhibits specific pathways, such as JNK pathway, in order to exert the protective role. These observations indicate that LanCL1 has protective effect against oxidative stressors, and that LanCL1 could be a novel therapeutic target for improving the efficiency of treating prostate cancer.

## Materials and methods

### Constructs

pPB-CAG-EBNXN vector was kind gifts from Sanger Institute. pPB-CAG-ires-Pac was generated as previously described^11,12^. pPB-CAG-LanCL1-ires-Pac was generated by ligating full length LanCL1 into the multiple cloning sites of pPB-CAG-ires-Pac.

### Cell lines and cell culture

BPH-1, LNCaP, PC-3, and DU145 cells were maintained in RPMI1640 supplemented with 10% FBS. All cells were supplemented with an antibiotic–antimycotic solution (100 units/ml penicillin, 0.1 mg/ml streptomycin, and 0.25 mg/ml amphotericin B) and grown at 37 °C in standard cell culture conditions (5% CO_2_, 95% humidity). Neo and LanCL1 stable LNCaP cells were obtained by co-transfection of LNCaP cells with pPB-CAG-LanCL1 and pCMVPBase. After 2 μg/ml puromycin (Amresco) screening for 2 weeks, stable cell lines were selected and identified by western blotting.

### Patient information

A group of 53 prostate cancer patients were recruited in for this study. Prostate cancer tissues were collected between 2011 and 2015 from Fudan University Huashan Hospital. These tissue samples were immediately snap-frozen in liquid nitrogen. The Clinical Research Ethics Committee of Fudan University Huashan Hospital approved the research protocols and written informed consents were obtained from the participants. Patients with a previous history of malignant tumors were excluded from this study.

### Tissue microarrays (TMAs) and immunohistochemistry (IHC)

Tissue microarrays (TMAs) were constructed as previously described^13^, and immunohistochemistry (IHC) was performed as described elsewhere^14,15^. In brief, slides were deparaffinized and heated in citrate buffer of pH 6 for antigenic retrieval. The primary antibody was LanCL1 (Proteintech, 1/600, 30 min). Immunohistochemistry was performed using the streptavidin-biotin-peroxidase method with diaminobenzidine as the chromogen (KitLSAB, Dakocytomotion, Glostrup, Denmark). Negative controls were obtained after the omission of the primary antibody or incubation with an irrelevant antibody.

LanCL1 staining was simultaneously examined by two blinded, independent observers (including one pathologist), and a consensus score was reached for each core. A positive reaction for LanCL1 was scored in four grade categories depending on the intensity of the staining, i.e., 0, 1, 2, and 3, and the percentage of LanCL1-positive cells was also scored in four groups: 0 (0%), 1 (1–33%), 2 (34–66%), and 3 (67–100%). In cases with discrepancies between duplicated cores, the higher score of the two tissues was taken as the final score. The sum of the intensity and percentage scores was used as the final staining score. The staining pattern was defined as follows: 0, negative; 1–2, weak; 3–4, moderate; and 5–6, strong^16^. The clinical information for the prostate cancer patients is included in the Table 1.

**Table 1:** Clinical characteristics of PCa patients and LanCL1 expression

Characteristics	Expression of LanCL1 in PCa		*P*-value
	Low (27)	High (26)	
*Age, years*			
≤65	13	7	0.111
>65	14	19	
*Gleason score*			
<7	8	2	**0.018**
7	12	11	
>7	6	12	
*Invasion of neuro bundle*			
Yes	3	6	0.2461
No	24	20	
*Invasion of seminal vesicle*			
Yes	0	6	**0.008**
No	27	20	

*P* < 0.05 was considered statistically significant. *χ*^2^-test was used. Bold values: The expression of LanCL1 in prostate cancer tissue was significant correlation with Gleason score and seminal vesicle invasion.

### TRAMP mice

All procedures involving laboratory animals conform to the Guide for the Care and the Use of Laboratory Animals published by the US National Institute of Health. Tramp mice are from The Jackson Laboratory and heterozygous for the PBTag transgene, were maintained in a C57BL/6 background. In this study, animals were killed at 25 weeks of age and IHC examination of prostate was performed.

### Xenograft growth of PC-3 cell tumors

Outbred male athymic mice (nu/nu) were kept in Fudan University. Six-week-old male athymic mice were inoculated subcutaneously with 100 μl of a mixture containing 1 × 10^7^ LanCL1 overexpressed or control PC-3 cells. Tumor growth was monitored weekly, and mice were killed after 4 weeks. Tumor sizes were measured recorded in mm^3^.

### siRNA transfections

We tested the individual set of three small interfering RNAs (siRNAs; GenePharma, China) against LanCL1 by western blotting. The two most-effective single siRNAs (GenePharma) against LanCL1 were used for further experiments.

For siRNA transfection in 6-well plates, 3 × 10^5^ LNCaP cells per well were subjected to reverse transfection with 100 nM LanCL1 or negative-control siRNA (GenePharma) using Lipofectamine 2000 transfection reagent (Invitrogen), following the manufacturer’s instructions.

### Antibodies and immunoblotting

Cells were lysed using 1× SDS loading buffer (50 mM Tris-HCl pH 6.8, 10% glycerol, 2% SDS, 0.05% bromophenol blue, and 1% 2-mercaptoethanol). Antibodies were purchased from the manufacturers as follows: anti-LanCL1 antibody (Proteintech), anti-4-HNE (Abcam), anti-E-cadherin (610181, BD Transduction Laboratories), anti-pSAPK/JNK (Thr183/Try185) (Cell Signaling Technology), anti-β-actin (Sigma–Aldrich), and anti-tubulin (Abcam). For immunoblot, proteins were separated by SDS–PAGE and transferred to polyvinylidene difluoride membranes (Millipore). HRP-conjugated secondary antibodies (Jackson Laboratories) and enhanced chemiluminescence system was used for signal detection. Protein was visualized using KODAK film machine or ChemiDoc XRS chemiluminescence detection and imaging system (Bio-Rad Laboratories).

### Cell proliferation (MTS) assay

The cells were seeded at 4000 cells/well (0.1 ml) in 96-well plates and incubated overnight at 37 °C for 6 days. At the end of the experiments, 20 μl of CellTiter 96 AQueous One solution reagent MTS [3-(4,5-dimethylthiazol-2-yl)-5-(3-carboxymethoxyphenyl)-2-(4-sulfophenyl)-2H-tetrazolium, inner salt] (Promega) in 100 μl of RPMI-1640 were added to each well, incubating cells for 1 h, based on the rate of color change. Cell viability was estimated by monitoring the absorbance at 490 nm using a SYNERGY HT microtiter plate reader (Bio-Tek).

### Wounding healing assay

PC-3 or LNCaP cells were seeded in 12-well dishes at a density of 1 × 10^5^ cells per well and grown to be confluent. The cell monolayers were scraped with a sterile white micropipette tip to create a denuded area of constant width. Cells were washed with PBS to remove cell debris, and supplemented with culture medium. The wound closure was monitored and photographed at indicated times after wounding.

### Real-time PCR (RT–PCR)

Total RNAs were extracted from cells using TRIzol reagent (Invitrogen). RNA was subjected to reverse transcription with reverse transcriptase as Manufacturer’s instructions (Fermentas). Quantitative real-time PCR (qRT–PCR) was performed using the Bio-Rad CFX96 system, and the relative gene expression was normalized to internal control as gapdh. Primer sequences for SYBR Green probes of target genes are as follows: LanCL1 (For: 5′-CCTTCAGGTGAACCAAGGAA-3′, Rev: 5′-AGATCACGTCAGCACACTGC-3′, GAPDH (For: 5′-TGATGACATCAAGAAGGTGGTGAAG-3′, Rev: 5′-TCCTTGGAGGCCATGTGGGCCAT-3′).

### Fluorescence-activated cell sorting analysis

Cells were allowed to grow to 70–80% confluence before trypsinization and resuspension in ice-cold buffer (30% phosphate-buffered saline, 70% ethanol) at a concentration of 1–5 × 10^6^ cells per ml for at least 1 h. The fixed cells were recovered by centrifugation (1000×*g*, 5 min), washed 3 times in PBS and resuspended in 1 ml PBS containing 8 mg RNaseA and 50 mg propidium iodide (PI). The PI stained samples were then incubated in the dark at room temperature for 30 min and assayed by FACS on a BD FACS Calibur with Cellquest software.

### Promoter reporter assays

The AP-1 and E-cadherin luciferase vectors were kindly provided by Dr. Qianyi Xiao. Promoter reporter assays were performed with the dual-luciferase reporter assay system (Promega) at 24 h after transfection, as described^17^.

### Determination of ROS level by fluorescence microtiter plate reader

Intracellular reactive oxygen species (ROS) were determined by fluorescence microscopy after cell labeling with DCFH-DA (7′-dichlorodihydrofluorescein diacetate, Beyotime). DCFH-DA is a nonfluorescent molecule, but once it enters into cells, it is deacetylated by cellular esterases and rapidly oxidized to highly fluorescent DCF (2′, 7′-dichlorodihydrofluoreskcein) by ROS. The observed fluorescence intensity is directly proportional to intracellular ROS levels. After treatment procedure, cells were stained with DCFH-DA (1 μM final concentration) and incubated for 15 min at 37 °C, washed with 1× PBS for three times and then visualized under fluorescent microtiter plate reader (Bio-tek) (excitation and emission wavelength of 495 and 528, respectively).

### Apoptosis signaling antibody array assay

**Fig. 1:**
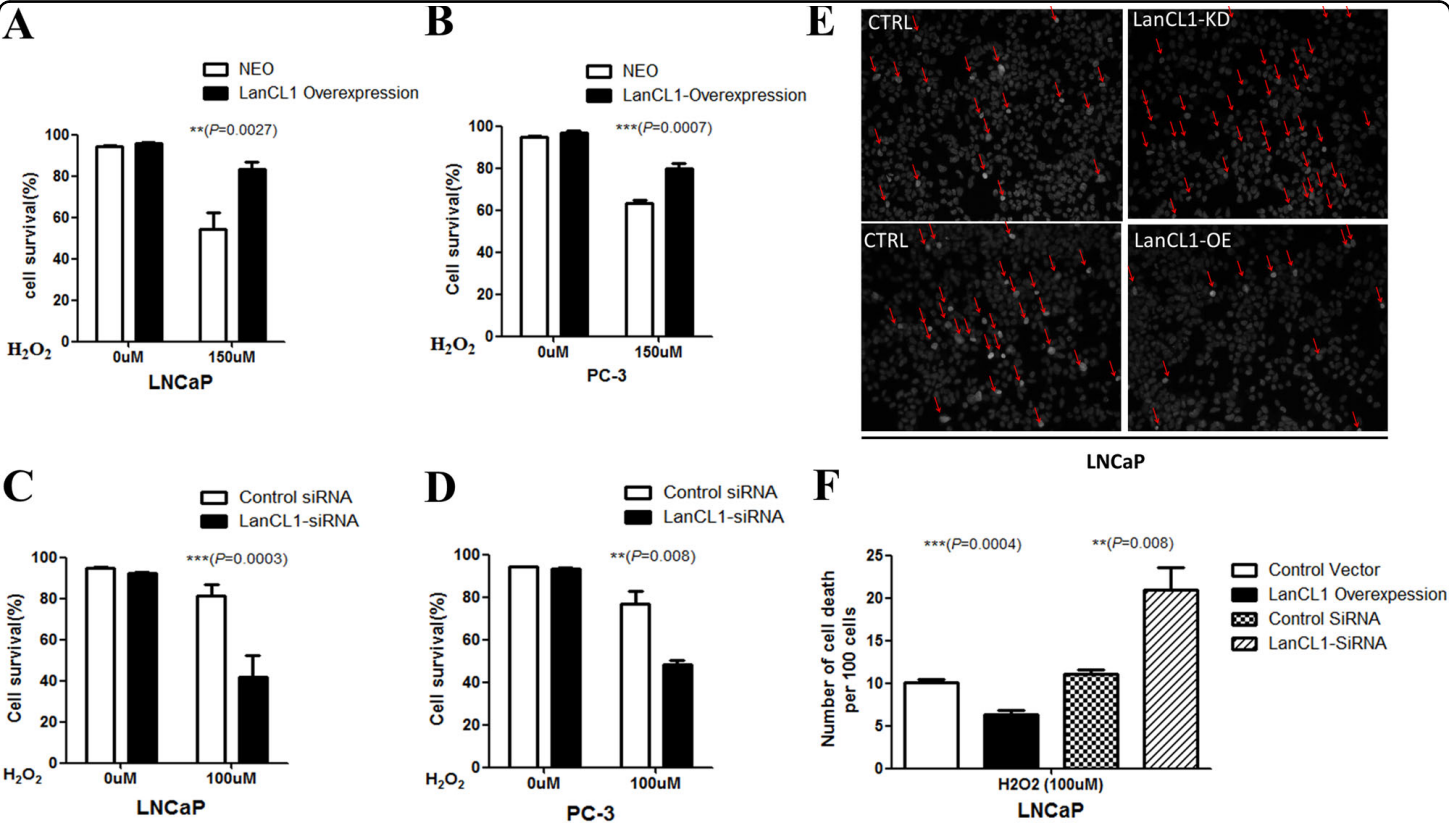
LanCL1 protects prostate cancer cells against ROS. **a**, **b** LanCL1 overexpression in LNCaP and PC-3 cells reduced cell death after 24 h treatment of 150 μM H2O2. Student’s *t*-test was performed for statistical significance analysis. *N* = 5. **c**, **d** LanCL1 knockdown increased LNCaP and PC-3 cells death induced by H_2_O_2_ (100 μM H_2_O_2_, 24 h treatment). Student’s *t*-test was performed for statistical significance analysis. *N* = 5. **e** Hoechst staining shows that downregulation of LanCL1 increased LNCaP cell death(indicated by arrows) induced by H_2_O_2_, while LanCL1 overexpression reduced cell death. **f** Quantitation of the Hoechst staining of LNCaP cell treated by H_2_O_2_. Student’s *t*-test was performed for statistical significance analysis. *N*>3

LNCaP-NEO and LNCaP-LanCL1 cells were seeded in 6-well plates and incubated overnight at 37 °C. Twenty-four hours later, H_2_O_2_ was added to the cultures at the final concentration of 100 μM for 4 h. A slide-based antibody array was used for simultaneous detection of 19 important signaling molecules involved in stress response and apoptosis, using a PathScan Stress and Apoptosis Signaling Antibody Array kit (Cell Signaling Technology, Danvers, MA) following the manufacturer’s instructions. Each of these molecules was detected in duplicate on the same array arranged as shown in Fig. 1b, and the identity of each numbered molecule listed in Fig. 1c. Briefly, total protein was extracted from tumor tissues using RIPA buffer, and diluted to 0.5 mg/mL in Array Diluent Buffer provided by the kit. The protein samples were incubated with the array antibodies overnight. A Detection Antibody Cocktail was added to the samples, followed by the addition of HRP-linked Streptavidin and substrate. Protein was visualized using ChemiDoc XRS chemiluminescence detection and imaging system (Bio-Rad Laboratories). The density of the spots was quantitated using Quantity One software (Bio-Rad Laboratories) and normalized to the α-tubulin levels. Each sample was done in duplicate.

### In vitro cell death and apoptosis assay

The cells were seeded in 12-well plates and incubated overnight at 37 °C. Twenty-four hours later, H_2_O_2_ was added to the cultures at the final concentration of 100 μM. Hoechst staining were performed Twenty-four hours after the H_2_O_2_ treatment. Cell death was quantified by Hoechst positive cells and statistical analysis was performed by using Student’s t-test.

### Rescue experiment

The cells were seeded in 96-well plates and incubated overnight at 37 °C. 24 h later, H_2_O_2_ was added to the cultures at the final concentration of 100 μM, with SP600125 (Beyotime) at 10 μM or DMSO (0.1% v/v). After treatment for 1 h, cell viability was estimated by MTS using a SYNERGY HT microtiter plate reader (Bio-Tek).

### Publically available gene expression data sets

In this article, we used RNA and protein expression data of LanCL1 in different human tissues and cancer cell lines from HPA portal through The Human Protein Atlas portal (http://www.proteinatlas.org/)^18^ and The Cancer Cell Line Encyclopedia (CCLE)^19^. All data are available directly online.

### Publically available clinical data sets

In this article, we used a cohort 150 human prostate tumors available with LanCL1 expression data and the deposition of a cohort of 176 prostate adenocarcinoma samples at the cBioPortal for Cancer Genomics (TCGA, Provisional)^20^ that allowed us to perform integrated analysis for single genes at this prepublication stage for other publications. Normalization procedures for gene expression intensity data are stated in individual data sets and related publications.

### Statistical analysis

The results are presented as the mean values ± SEM. The association between LanCL1 staining and the clinicopathologic parameters of the prostate cancer patients were evaluated by a *χ*^2^-test. Differences between groups were estimated using the *χ*^2^-test, Student’s *t*-test, the Mann–Whitney *U*-test, repeated-measures analysis of variance test. A value of *P* < 0.05 was considered statistically significant.

## Results

### LanCL1 expression correlates with human prostate cancer progression

**Fig. 2:**
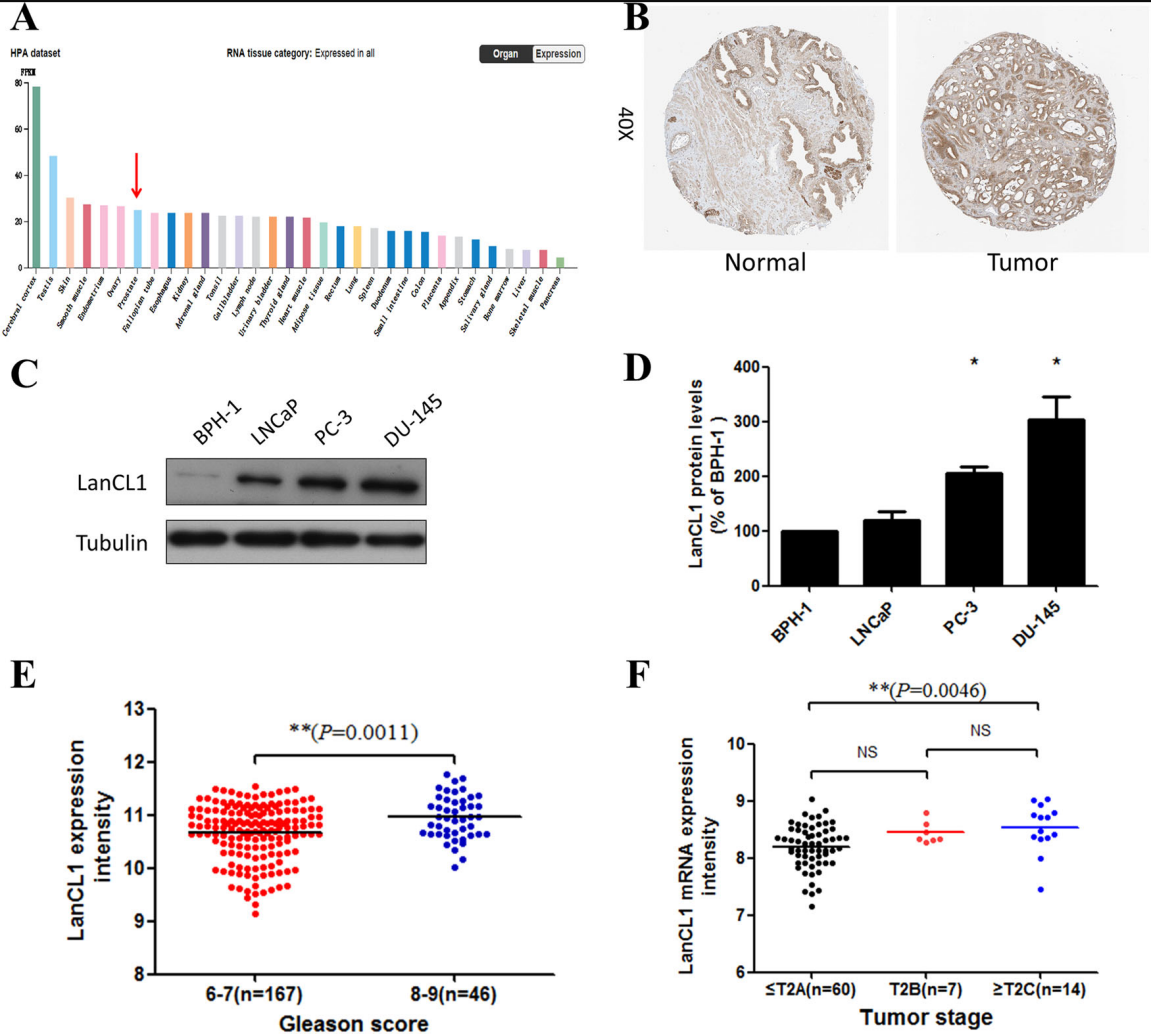
Associations between LanCL1 expression and prostate cancer risk and severity. **a** Data from The Human Protein Atlas portal show LanCL1 expression in different tissues. Prostate is indicated by red arrow. **b** IHC data from The Human Protein Atlas portal show LanCL1 expression is higher in prostate cancer tissues than in normal tissues. **c**. Western blotting was used to detect the LanCL1 expression in human prostate cell lines. Tubulin served as the control. **d** Quantitation of relative expression of LanCL1 protein in different prostate cancer cell lines. **e**, **f** Increased expression markedly correlates with high Gleason score and tumor stage, data from the TCGA data set (queried from cBioPortal). LanCL1 expression is exon-normalized signal intensity. Mann–Whitney *U*-tests were performed to assess statistical significance for the comparisons between groups

To investigate the role of LanCL1 in prostate cancer, we first investigated the expression of LanCL1 in different tissues. Data from The Broad-Novartis Cancer Cell Line Encyclopedia (CCLE) and The Human Protein Atlas portal showed that the expression of LanCL1 is higher in prostate cancer tissues and human prostate cancer cell lines, which suggests that LanCL1 may be upregulated in prostate cancer cells. Consistently, LanCL1 also expresses in normal prostate tissue (data from HPA, Fig. 2a, b, Supplementary Figure 1A).

Next, we assessed the level of LanCL1 protein in various prostate cancer cell lines and found that LanCL1 was highly expressed in the PC-3 and DU145 metastatic prostate cancer cells, but we detect a relatively much lower LanCL1 expression in the benign or weakly metastatic prostate cancer cells including BPH-1 and LNCaP (Fig. 2c, d). Furthermore, we investigated the relationship between LanCL1 expression and the clinic pathological characteristics in the TCGA using cBioPortal for Cancer Genomics^20^. We found that the upregulation of LanCL1 significantly correlate with higher Gleason score and more aggressive tumor stage (Fig. 2e, f).

**Fig. 3:**
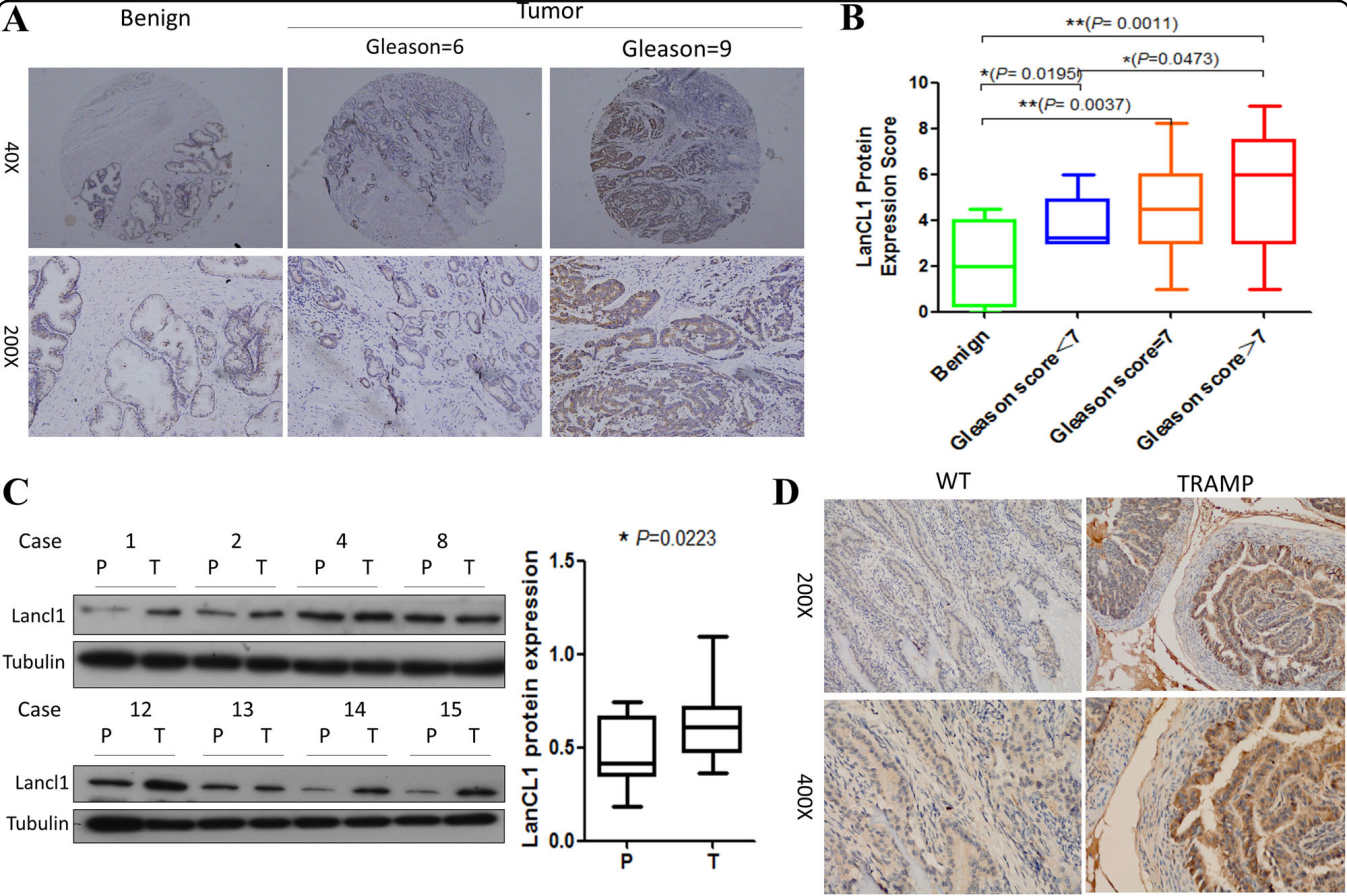
Protein level of LanCL1 is upregulated in prostate tumors. **a** Representative immunohistochemistry of LanCL1 on benign prostatic epithelia (benign) and prostate cancer tissues (Gleason score 6 or 9). Student’s t-test was performed for statistical significance analysis. **b** Quantitation of protein expression of LanCL1 in benign or prostate cancer tissues. **c** Western blot analysis of LanCL1 expression in 15 pairs of nontumor tissues (P) and PCa tissues (T). Student’s t-test was performed for statistical significance analysis. *N* = 15. **d** Immunohistochemical staining for LanCL1 protein in the dorsolateral prostate of WT and TRAMP mice

To further confirm LanCL1 protein expression levels in prostate cancer progression, we examined expression of LanCL1 by IHC in prostate cancer tissues. As shown in Fig. 3a, the expression of LanCL1 is significantly higher in prostate cancer tissues compared with benign prostatic epithelia. Importantly, immunostaining for LanCL1 was significantly stronger in more aggressive prostate cancer tissues, as indicated by Gleason score (Fig. 3b). We next examined LanCL1 expression in 15 cases of prostate tumor and matched nontumor tissues by western blot. Results also revealed that LanCL1 expression in tumors were higher than that of matched nontumor tissues (Fig. 3c). Furthermore, immunohistochemical analysis of prostate tissue sections for LanCL1 in WT and TRAMP mice at 25 weeks of age showed that LanCL1 expression level was upregulated in the TRAMP prostate cancer tissues (Fig. 3d).

All these results revealed that LanCL1 expression level correlates with advanced tumor stage and poor prognosis in prostate cancer patients, suggesting that LanCL1 is likely involved in the tumorigenesis and progression of prostate cancer.

### LanCL1 protects prostate cancer cells from death induced by ROS

**Fig. 4:**
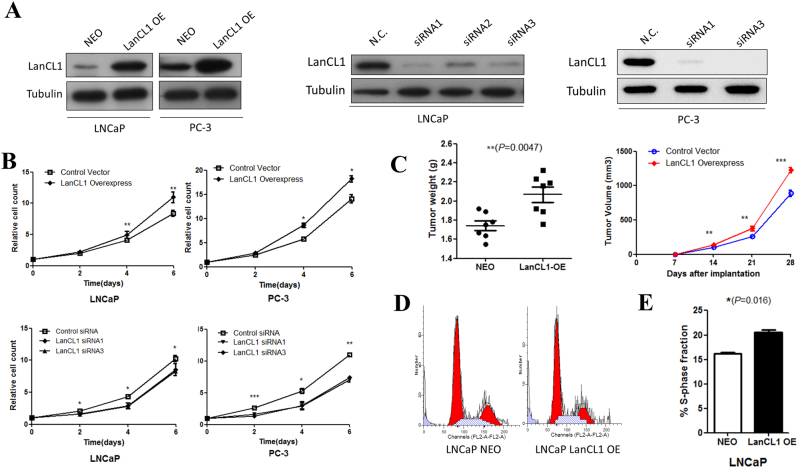
High LanCL1 expression promotes proliferation of prostate cancer cells in vitro and in vivo. **a** Stably transfected LNCaP cells and PC-3 cells interfered with LanCL1 were established. LanCL1 was knockdown by siRNA as indicated. The efficiency of knockdown were examined by western blotting. Tubulin served as the control. **b** Cell proliferation was measured at the indicated time points by MTS colorimetric assay (absorbance at 490 nm (OD490). Student’s *t*-test was performed for statistical significance analysis. *N*>3. *P<0.05, **P<0.01, ***P<0.001 **c** Tumor growth in LanCL1 overexpressed PC-3 cells and control cells was investigated by xenograft tumor models. Student’s t-test was performed for statistical significance analysis. *N* = 7. **P<0.01, ***P<0.001. **d** The cell cycle distribution was analyzed by fluorescent-activated cell scanning (FACS) in LanCL1 overexpressed LNCaP cells. **e** Column chart indicates the mean and SEM of the S-phase fraction for each cell. Student’s t-test was performed for statistical significance analysis. *N* = 5

The above data suggest that LanCL1 is potentially involved in the regulation of prostate cancer progression. To test the biological relevance of LanCL1 in prostate cancer cells, we examined the effect of LanCL1 overexpression and knocking down on cellular phenotypes. We chose LNCaP and PC-3 cell lines and established two stably expressing LanCL1 cell lines (LNCaP-LanCL1 and PC-3-LanCL1), and knocked down LanCL1 expression by siRNA. Western blotting revealed higher expression of LanCL1 in comparison to that in a syngeneic control subline (LNCaP-NEO and PC-3-NEO), and much lower LanCL1 gene expression in knocking down cell lines (Fig. 4a).

We first examined the effect of LanCL1 on cell proliferation. Results showed that LanCL1 promotes prostate cancer proliferation (Fig. 4b). Consistently, by successful construction of a xenograft tumor model of PC-3 cells, we confirm that upregulation of LanCL1 expression promotes the growth of tumor in vivo (Fig. 4c). In further exploration, we found a slight higher proportion of the cells in S-phase in comparison with control cells (Fig. 4d, e, Supplementary Figure 1B). Furthermore, LanCL1 does not contribute to the cell migration of prostate cancer cells and EMT progression (Supplementary Figures 2A, B).

Previous studies have shown that LanCL1 serves as an antioxidant gene in brain neurons, which catalyzes the formation of thioether products and protects cells against oxidative stress^7^. To examine whether LanCL1 plays the same role in prostate cancer cells, we cultured wild-type prostate cancer cells LanCL1 overexpression or knockdown cells. In normal medium, all cells grow well without apparent cell death. To induce ROS, the cells were treated with oxidative stress-inducing agents H_2_O_2_^21^. As expected, LanCL1 overexpression resulted in resistance to H_2_O_2_-induced cell death (Fig. 1a, b), while LanCL1 loss led to increased cell death in LNCaP and PC-3 cells (Fig. 1c, d). Furthermore, we also examined cell apoptosis by Hoechst staining. Apoptotic cells always show a homogeneous intense staining of the nucleus or nuclear fragments with Hoechst staining. As shwon in Fig. 1e, f and Supplementary Figure 1C, downregulation of LanCL1 resulted in increased LNCaP and PC-3 cells death, while LanCL1 overexpression showed opposite effect. Taken together, these findings indicated that LanCL1 could protect prostate cancer cells from high-level H_2_O_2_-induced cell death.

### LanCL1 does not mitigate oxidative stress in prostate cancer cells

**Fig. 5:**
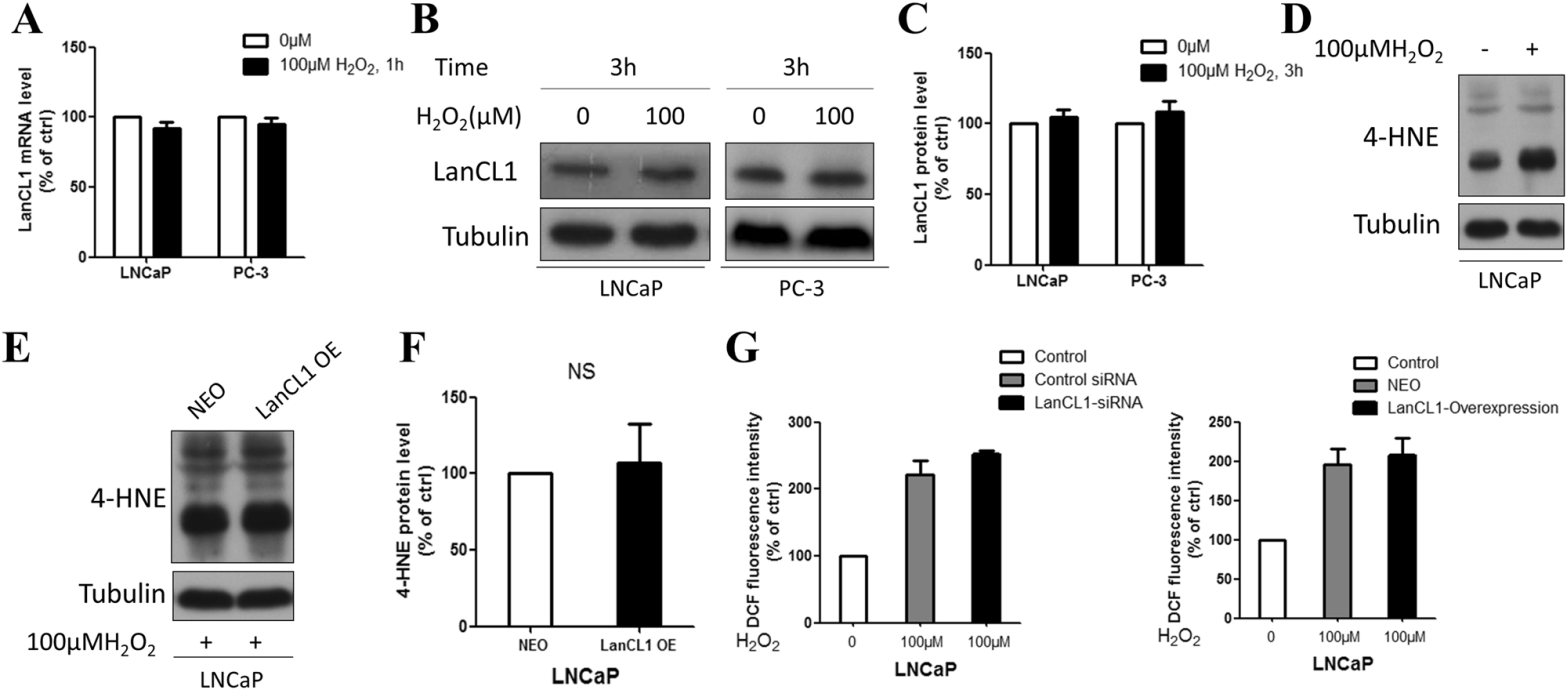
LanCL1 does not mitigate oxidative stress. **a** qRT–PCR shows no induction of LanCL1 mRNA in H_2_O_2_-treated (100 μM, 1 h) LNCaP cells. Student’s *t*-test was performed for statistical significance analysis. *N* = 3. **b**, **c** Western blots and quantification show that H_2_O_2_-treatment did not induce LanCL1 protein expression. Student’s t-test was performed for statistical significance analysis. *N* = 5. **d**–**f** Western blots and quantification show an increase in the level of 4-HNE in the H_2_O_2_-treated (100 μM, 1 h) LNCaP cells, but LanCL1 overexpression did not influence the level of 4-HNE. Western blots show that LanCL1 overexpression in LNCaP cells is not more resistant to H_2_O_2_-induced 4-HNE accumulation. Student’s t-test was performed for statistical significance analysis. *N* = 5. **g** ROS determination by using a fluorescent probe DCFH-DA in LNCaP and PC-3 cells untreated and treated with H_2_O_2_ (100 μM, 1 h). Student’s t-test was performed for statistical significance analysis. *N* = 3

To investigate the potential mechanism of LanCL1 protecting LNCaP cells from cell death, we first examined whether LanCL1 mitigates oxidative stress, as previous study showed. To elucidate this question, we first detected whether H_2_O_2_ could induce LanCL1 expression. By qRT–PCR and western blotting, we found no significant LanCL1 mRNA and protein levels upregulation after H_2_O_2_ treatment in LNCaP or PC-3 cells (Fig. 5a–c). Furthermore, we used DCFH-DA and 4-HNE as indicators to show the oxidative stress level in prostate cancer cells. Previous researches have described the use of Dichloro-dihydro-fluorescein diacetate (DCFH-DA) as a fluorometric assay for hydrogen peroxide^22^, which makes dichlorofluorescin (DCFH) serving as a probe to evaluate intracellular hydrogen peroxide formation. 4-hydroxy-2-nonenal (4-HNE) is an indicator of lipid peroxidation and oxidative stress because it is generated in the process of oxidation of polyunsaturated fatty acids^23^. As expected, 4-HNE protein level in LNCaP cells increased dramatically after treated by H_2_O_2_ (Fig. 5d), so does the increased DCF fluorescence (Fig. 5g). To our surprise, however, overexpressing LanCL1 in LNCaP or PC-3 cells did not affect the accumulation of 4-HNE and DCF fluorescence induced by H_2_O_2_ treatment (Fig. 5e–g, Supplementary Figure 2C, D). These results indicated that LanCL1 does not exert its protective effect by mitigating oxidative stress in prostate cancer cells.

### LanCL1 protects prostate cancer cells from oxidative stress via suppression of JNK pathway

**Fig. 6:**
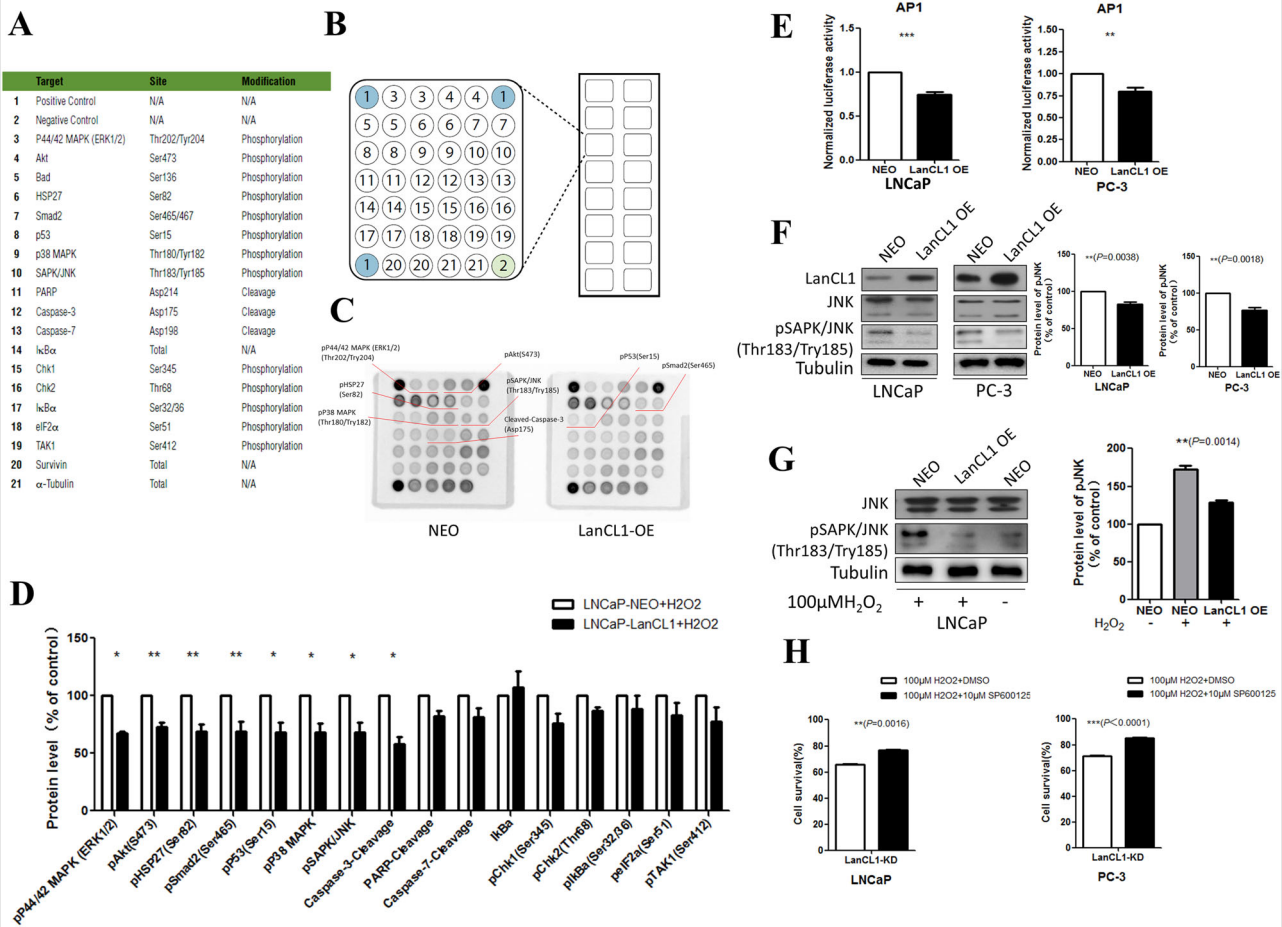
LanCL1 protects prostate cancer cells via suppression of JNK pathway. **a**, **b** Modulation of apoptosis related signaling proteins using antibody array assay. Total protein was extracted from LNCaP-NEO and LNCaP-LanCL1 cells. A slide-based antibody array was used for simultaneous detection of 19 signaling molecules involved in stress response and apoptosis using a PathScan Stress and Apoptosis Signaling Array kit. Each protein was arranged in duplicate. **c** The proteins with changes concentration included pP44/42 MAPK (ERK1/2) (Thr202/Try204), pAkt(S473), pHSP27(Ser82), pSmad2(Ser465), pP53(Ser15), pP38 MAPK(Thr180/Try182), pSAPK/JNK(Thr183/Try185) and Cleaved-Caspase-3(Asp175), which were indicated on the images, and quantified. **d** The concentrations of the protein in **c** were quantified. Student’s *t*-test was performed for statistical significance analysis. *N* = 3. Data are presented as mean ± SEM. *P<0.05, **P<0.01, ***P<0.001. **e** Luciferase assay shows that overexpression of LanCL1 reduced AP-1 transcription. Student’s t-test was performed for statistical significance analysis. *N* = 3. ***P<0.001 **f** Western blotting indicated the protein level of pSAPK/JNK(Thr183/Try185) in overexpression LNCaP and PC-3 cells. Student’s *t*-test was performed for statistical significance analysis. *N* = 3. **g** Western blotting indicated the protein level of pSAPK/JNK(Thr183/Try185) in overexpression LNCaP cells after 100 μM H_2_O_2_ for 2 h. Student’s t-test was performed for statistical significance analysis. *N* = 3. H. SP600125 partially rescued the cell death caused by ROS in LanCL1 knockdown LNCaP cells. Student’s t-test was performed for statistical significance analysis. *N* = 5

After excluding the possibility that LanCL1 protects prostate cancer cells through mitigating oxidative stress, we wonder how LanCL1 makes it. We supposed that LanCL1 might exert the role by influencing the pathways downstream of oxidative stimulation. To verify this hypothesis, we used a PathScan Stress and Apoptosis Signaling Array kit to find out the specific downstream pathway involved. As Fig. 6a, b indicated, we detected 19 signaling molecules involved in stress response and apoptosis. Results showed decreased protein level of several pathways in LanCL1 overexpression group (Fig. 6c, d).

Among all the pathways above, c-Jun N-terminal kinase (JNK) pathway, whose activity is indicated by pSAPK/JNK (Thr183/Try185) protein level, has been confirmed to be implicated in oxidative stress pathway^24,25^. We are interested in whether LanCL1 influences JNK pathway activity. To determine this question, we first examined AP-1 activity, a transcription factor regulated by JNK signaling, by a dual-luciferase reporter assay system (Promega). We found that LanCL1 overexpression significantly inhibited AP-1-directed luciferase activity (~30%, Fig. 6e), indicating downregulation of JNK signaling. To examine the connection between LanCL1 and p-JNK in prostate cancer, we determined LanCL1 and p-JNK expression in different prostate cancer cell lines. The expression of pSAPK/JNK (Thr183/ Try185) was found to be high in the low LanCL1-expressing prostate cancer cell line LNCaP, whereas the expression was low in high LanCL1-expressing PC-3 and DU145 cells, indicating the inverse correlation between LanCL1 and p-JNK expression levels (Supplementary Figure 2E).

To confirm whether LanCL1 influences JNK pathway activity, we detected pSAPK/JNK (Thr183/Try185) expression in LanCL1 overexrepssing cells. Western blotting showed decreased pSAPK/JNK (Thr183/Try185) expression level in both LNCaP-LanCL1 and PC-3-LanCL1 (Fig. 6f). In addition, we also observed decreased level of pSAPK/JNK (Thr183/Try185) in LNCaP-LanCL1 and PC-3-LanCL1 cells, and increased level of pSAPK/JNK (Thr183/Try185) in LanCL1 loss LNCaP and PC-3 cells upon H_2_O_2_ treatment for 2 h, in comparison with control cells (Fig. 6g, Supplementary Figure 3).

To further confirm whether JNK pathway involves in LanCL1 exerting its role of protecting cell viability, we next asked whether JNK inhibition by SP600125, a new JNK inhibitor^26^, could rescue the increased cell apoptosis induced by H_2_O_2_ in LanCL1 knockdown cells. As shown in Fig. 6h, specifically blocking JNK activity in LanCL1 knockdown LNCaP and PC-3 cells by SP600125 significantly attenuated cell death caused by H_2_O_2_ treatment. All these results suggest that LanCL1 protects prostate cancer cells from H_2_O_2_-induced cell death mainly through suppressing JNK signaling activity.

## Discussion

Prostate cancer (PCa) is still the most common malignant tumor and the second most common cause of cancer-related deaths among men in the developed world^1^. Although only about 10% of men with newly diagnosed PCa have locally advanced disease, most androgen-dependent disease will develop to castration-resistant prostate cancer (CRPC) after receiving castration or anti-androgen therapies (androgen deprivation therapy), in which stage less treatment works. The prognosis of locally advanced or metastatic PCa remains unsatisfactory. There is an urgent need to find out the mechanisms of disease and to develop new therapeutic strategies.

ROS and oxidative stress play complicated and important roles in cancer initiation and development. In cell microenvironment, oxidative stress always results from the imbalance of reactive oxygen species (ROS) and enzymes that help mitigate cellular levels of ROS. Overload of ROS leads to cumulative damage to lipids, proteins, and DNA. Several lines of evidence have suggested that oxidative stress and its cumulative impact on DNA damage contributes the major aging-associated influences on prostate carcinogenesis^10^. Such evidence also indicates that prostate may be more vulnerable to oxidative stress, as major antioxidant enzymes are reduced in human PIN and prostate cancer, while there is an increase of the oxidized DNA products^27^.

Some other studies have shown that in prostate cancer progression, low concentrations of ROS promote prostate cancer development by stimulating cell proliferation and migration through activation of multiple pathways, while high level leads to apoptosis^28–30^. Especially, in the progression of CRPC, castration-induced oxidative stress may contributes to aberrant androgen receptor (AR) signaling and other pathways activation, which results in a gain of castration resistance^31,32^.

CRPC is generally associated with very poor prognosis and is sensitive to only a little chemotherapeutic drugs, among which docetaxel is currently an important treatment option for patients. Recent evidence have shown that docetaxel antitumor activity partially results from its ability to trigger high-level ROS formation in prostate cancer cells, thus inducing DNA, protein and cell membrane damage. However, nearly all patients with CRPC treated with docetaxel eventually become refractory. Therefore, the ability of cancer cell in defensing ROS becomes a potential drug resistance mechanism in PCa^33^. All in all, ROS plays quite important and complicated roles in prostate cancer, which makes ROS and genes related to ROS become potential therapeutic target.

The c-Jun NH2-terminal kinases (JNKs) are an evolutionarily conserved sub-group of mitogen-activated protein (MAP) kinases. Members in this pathway function in a cell context-specific manner to integrate signals that affect cell proliferation, differentiation, survival and migration^24^. JNK has been confirmed to be involved in the development of various tumor types, including prostate cancer. PTEN loss leads to AKT activation and increased JNK activity in human prostate cancer cell lines and human clinical prostate cancer samples, which promotes prostate cancer development^34^. What’s more, researchers also confirmed JNK as a core mediator of ROS and oxidative stress inducing apoptosis in various cell types, including prostate cancer cells. Results have showed that ROS promotes the activation of JNK and DNA damage response and induces growth arrest/senescence to apoptosis in prostate cancer cell. Activated JNK regulates AP-1 and downstream apoptosis pathways, such as p53, PARP-1 activation^25,35–37^. Therefore, JNK pathway is a core contributor in ROS inducing prostate cancer apoptosis and is a potential therapeutic target.

LanCL1 is a new gene that can help cells deal with ROS. It can bind zinc ion and GSH and is essential for mitigating neuronal oxidative stress by catalyzing the formation of thioether products^5–7^. In the present study, we analyzed the expression of LanCL1, and reveal the function of LanCL1 in protecting prostate cells from ROS and oxidative stress. Online data sets and immunohistochemical analysis of clinically localized prostate cancer cases demonstrated that prostate cancer cells were positive for LanCL1, whereas weak or no staining of LanCL1 was observed in non-tumor samples. Similarly, the more aggressive prostate cancer cell lines express higher level of LanCL1. Furthermore, we also found the association between LanCL1 expression and Gleason score and tumor stage. All these results indicate that LanCL1 may serve as an oncogene in prostate cancer.

To further research the function and mechanism, in the study, we overexpressed or knocked down LanCL1 in prostate cancer cells and found that LanCL1 promotes cell proliferation by influencing cell cycle. As what happens in neuron cells, LanCL1 also protects prostate cancer cells from high-level ROS, nevertheless, the mechanism is not the same. Cells with different expression levels of LanCL1 showed different sensitivity to H_2_O_2_ treatment. LNCaP cells, which showed decreased LanCL1 expression, were more sensitive to H_2_O_2_ treatment. LanCL1 does not help mitigate ROS level in prostate cancer cell, but suppresses the pathways downstream of oxidative stress. JNK pathway was found to be the main contributor. These results suggest that LanCL1 plays an important role in prostate cancer progression at least by protecting cells from high level of ROS through suppressing JNK pathway, while the roles of other pathway are needed further research.

To further understand the mechanisms of LanCL1 and pathways implicated, we will work on in the following investigation. We observed increased expression of LanCL1 during prostate cancer development. To dig out the possible mechanism of such increased gene expression, we analyzed cases with available mutation, copy number variation (CNV) data and mRNA level of LanCL1 in the TCGA PCa data set using cBioPortal for Cancer Genomics. Results indicated that deletion or amplification of LanCL1 is quite infrequent (<2%, data not shown) and that the upregulation of LanCL1 is not linked with a highly unstable genomic region. We put forward the hypothesis that the upregulation of LanCL1 expression might due to function of some transcription factors or some other mechanisms, which we will further investigate in future work.

Besides, the molecular links among LanCL1 expression and p-JNK downregulation is still an interesting issue. Many signaling pathways have been confirmed to be implicated in regulating JNK pathway in prostate cancer^28,38,39^. Among them, AKT pathway is a potential target of LanCL1, which has been shown to influence JNK activity in H_2_O_2_-induced cell death in prostate cancer^40–42^. Previous study showed that LanCL1 knockdown increases the level of phosphorylation in AKT (S473) and Erk in HEK293 cells^43^. It is possible that LanCL1 inhibits JNK activity via AKT and Erk pathways, but it is also probably not the case. Molecular mechanisms are always dependent on cell context. Therefore, we will investigate the mechanism how LanCL1 inhibits JNK pathway activity in prostate cancer in the following study.

In summary, we found upregulated LanCL1 expression in prostate cancer tissues. LanCL1 enhances cancer cell proliferation, and protects cells from ROS via suppressing JNK signaling pathway, mainly. Although we showed the important role of LanCL1 in prostate cancer progression, the underlying mechanism is still largely unknown. In consideration of the important role of ROS in CRPC development and chemotherapeutic drugs resistance, there is an urgent need for further study of digging out other function of LanCL1 in prostate cancer. Meanwhile, we confirmed that LanCL1 could stimulate cancer cell proliferation, making us deliberate whether it may contribute to cancer progression through some mechanisms other than ROS. Therefore, further study would clarify the biological significance of LanCL1 in prostate cancer.

Our study contained one limitation. In our study, we can only make an investigation that LanCL1 protects cells from ROS via suppressing JNK signaling pathway. The possible molecular links among LanCL1 expression and p-JNK downregulation needs further study.

## Electronic supplementary material

Sup Figure 1

Sup Figure 2

Sup Figure 3

Sup Figure Ligends
