# Supplementary figures and images for: LanCL1 protects prostate cancer cells from oxidative stress via suppression of JNK pathway
Source: Cell Death Dis. 2018 Feb 7;9(2):197. doi: 10.1038/s41419-017-0207-0 (PMC5833716; doi:10.1038/s41419-017-0207-0)

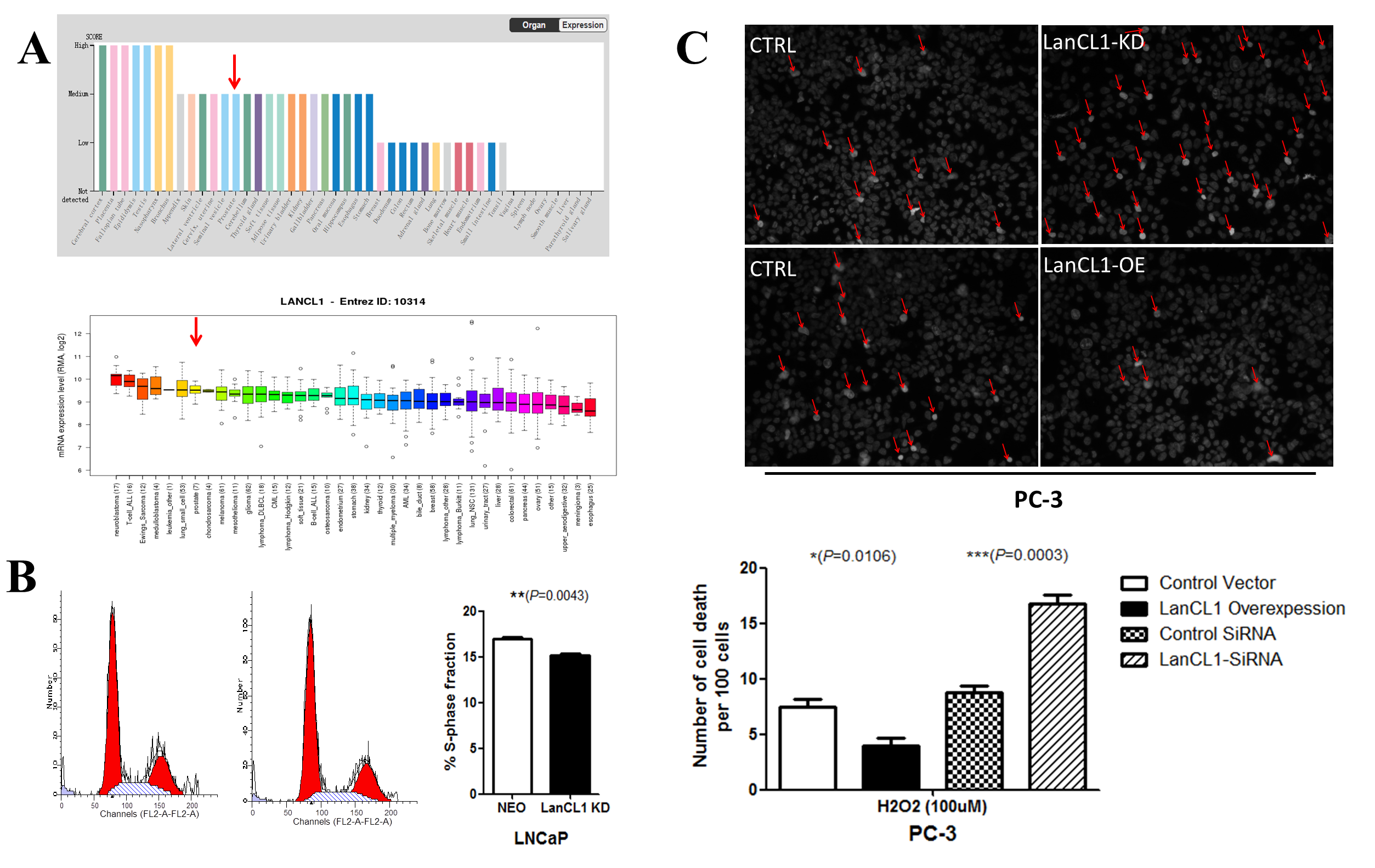

Supplement: Supplementary file 1 — Sup Figure 1 [file 41419_2017_207_MOESM1_ESM.tif]

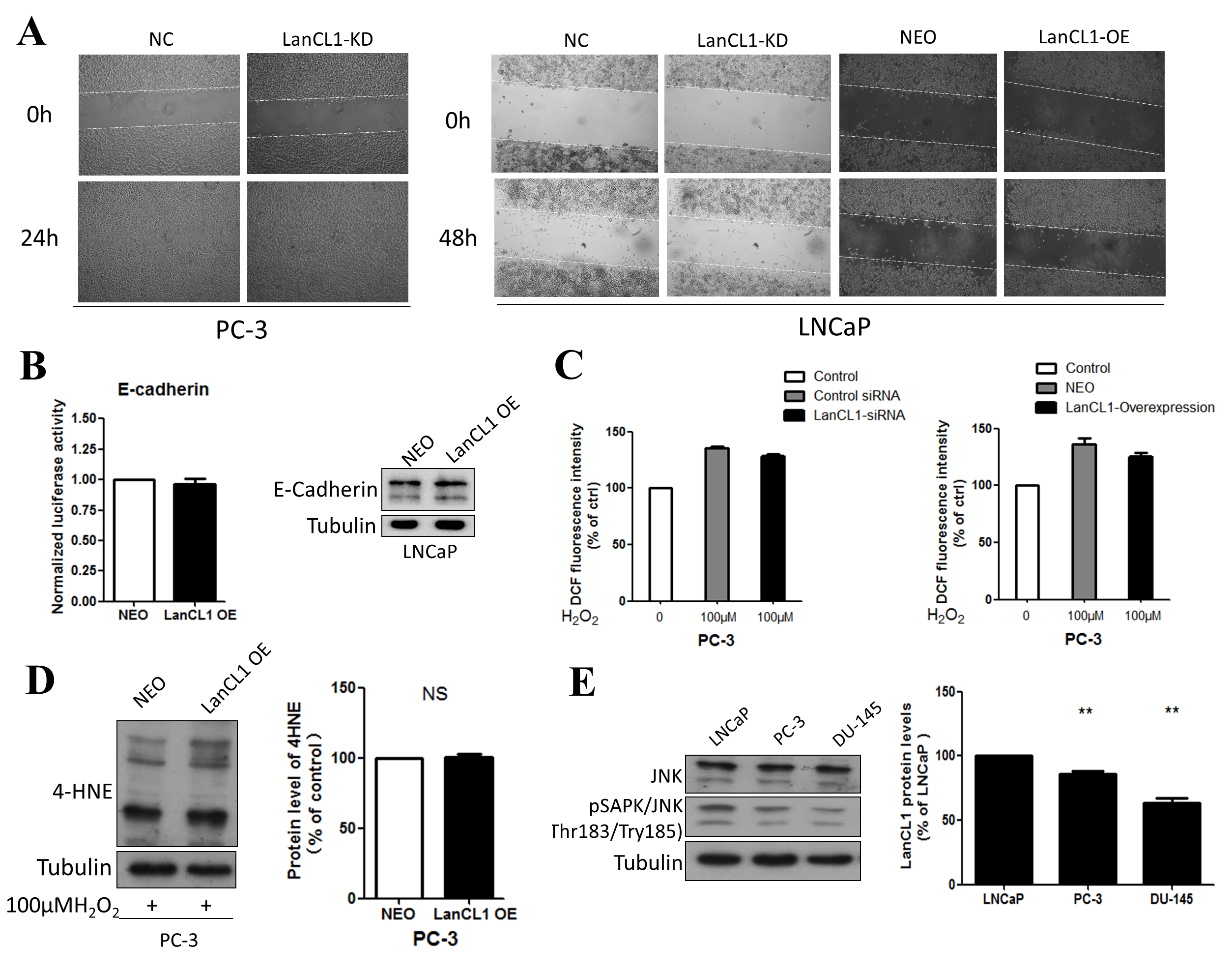

Supplement: Supplementary file 2 — Sup Figure 2 [file 41419_2017_207_MOESM2_ESM.tif]

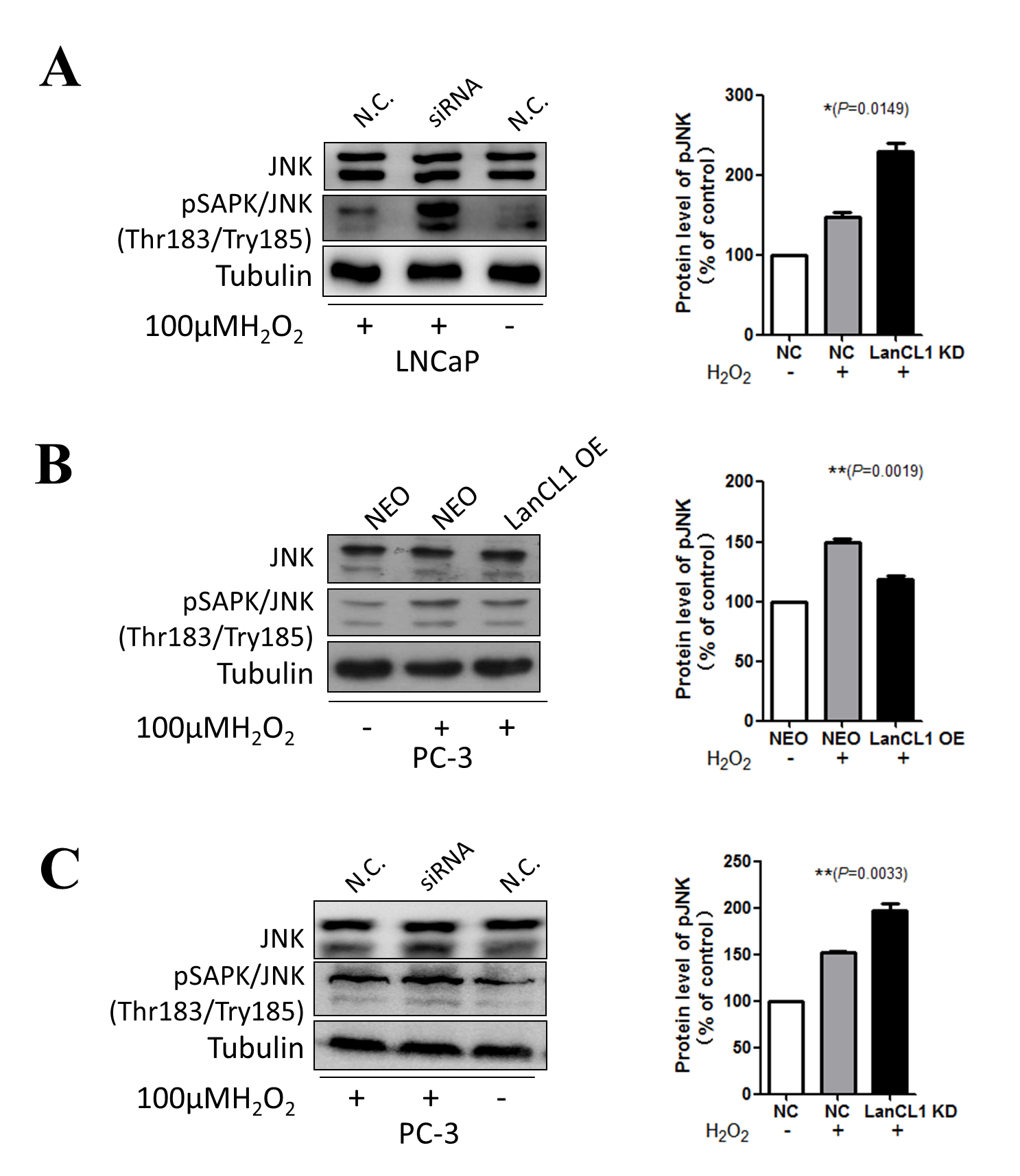

Supplement: Supplementary file 3 — Sup Figure 3 [file 41419_2017_207_MOESM3_ESM.tif]
