# Supplementary material for: LanCL1 protects prostate cancer cells from oxidative stress via suppression of JNK pathway
Source: Cell Death Dis. 2018 Feb 7;9(2):197. doi: 10.1038/s41419-017-0207-0 (PMC5833716; doi:10.1038/s41419-017-0207-0)
Supplement: Supplementary file 4 — Sup Figure Ligends [file 41419_2017_207_MOESM4_ESM.docx]

**Supplementary Figure Legends**

**Supplementary Figure 1.**

A. Data from The Human Protein Atlas portal and the Broad Cancer Cell Line Encyclopedia portal show LanCL1 expression in different tissues and in various cancer cell lines. Prostate is indicated by red arrow. The figure has been adapted from the HPA and CCLE portal.

B. The cell cycle distribution was analyzed by fluorescent-activated cell scanning (FACS) in LanCL1 knocked down LNCaP cells. Column chart indicates the mean and SEM of the S-phase fraction for each cell. Student’s t test was performed for statistical significance analysis. N=5.

C. Hoechst staining shows that downregulation of LanCL1 increased PC-3 cell death(indicated by arrows) induced by H_2_O_2_, while LanCL1 overexpression reduced cell death. Quantitation of the Student’s t test was shown. Student’s t test was performed for statistical significance analysis. N＞3.

**Supplementary Figure 2.**

A. Wound healing assay of the LNCaP overexpressed stable cells, knock-down cells and PC-3 LanCL1 knock-down cells. Cell images were taken at the indicated time points.

B. Luciferase assay and western blotting show that overexpression of LanCL1 does not influence E-cadherin transcription and protein level. Student’s t test was performed for statistical significance analysis. N=3.

C. ROS determination by using a fluorescent probe DCFH-DA in LNCaP and PC-3 cells untreated and treated with H_2_O_2_ (100μM, 1h). Student’s t test was performed for statistical significance analysis. N=3.

D. Western blots show that LanCL1 overexpression in PC-3 cells is not more resistant to H_2_O_2_-induced 4-HNE accumulation. Student’s t test was performed for statistical significance analysis. N=3.

E. Western blotting indicated the protein level of pSAPK/JNK (Thr183/Try185) in LNCaP, PC-3 and DU145 cells. Student’s t test was performed for statistical significance analysis. N=3.

**Supplementary Figure 3.**

Western blotting indicated the protein level of pSAPK/JNK(Thr183/Try185) in LanCL1 KD LNCaP (A), LanCL1-PC-3 (B) and LanCL1 KD PC-3 (C) cells after 100μM H_2_O_2_ for 2 hours. Student’s t test was performed for statistical significance analysis. N＞3.
